# Is a Small-Pox Hospital a Nuisance?

**Published:** 1898-04-16

**Authors:** 


### The Institutional Workshop.

### IS A SMALL-POX HOSPITAL A NUISANCE ?

Whatever we may think of the theory of the aërial convection of the infection of small-pox, there can be no doubt that the very basis on which it stands is the "fact" that those dwelling in the near neighbourhood of a small-pox hospital necessarily run a greater risk of catching the disease than those living at a distance. Hence the great importance of the judgment delivered by Mr. Justice Romer in the case of Harrop v. the Mayor, &c., of Ossett, for he said that, "Looking at the site of the present hospital, he found that it was on high, well-ventilated, and very open ground; and, taking into account the small extent of the building, he was not satisfied that there was a substantial source of danger either to the plaintiffs or their employés." Now it must be borne in mind that, according to the evidence, the hospital in question was only about 70 yards distant from a dwelling-house occupied by its owner, 100 yards from a warehouse, and 250 yards from a mansion, all of which were thus well within a quarter of a mile of it—were, in fact, within that inner zone which, according to those who believe in the aërial convection of small-pox, is of necessity far more likely than other parts to be infected with that disease. The position in which the question now stands is interesting. According to the believers in aërial convection, including, of course, the Local Government Board, whether a small-pox hospital be well conducted or ill conducted, whether its patients be few or many, so long as the cases treated within it are acute, those people who dwell in the neighbourhood will be more likely to become infected than if the hospital were not there, and this by means of an agency entirely beyond control. Moreover, these authorities are so convinced of the reality of the danger that they would not sanction, if they could help it, the erection of a small-pox hospital in the neighbourhood of habitations. Yet, the judge, after hearing all the evidence, says that he is not satisfied that such a hospital would be a substantial source of danger or a legal nuisance even to people within the quarter-mile zone It is unnecessary to point out how great is the divergence between the legal and the official views. The judgment would seem in direct opposition to the very fact upon which the theory of aërial convection is based; that is the danger of infection which hangs over those dwelling near a small-pox hospital.

